# Number blindness in human vision

**DOI:** 10.3758/s13414-025-03113-7

**Published:** 2025-06-19

**Authors:** James Negen

**Affiliations:** Psychology Department, Liverpool John Moores University, Liverpool, UK

**Keywords:** Visual perception, Cognitive neuroscience, Signal detection theory

## Abstract

There is an ongoing controversy over whether human vision first estimates area and number, deriving our sense of density via division, or if it first estimates area and density, deriving our sense of number via multiplication. If number and area are both primary independent dimensions of visual perception then we should observe cross-magnitude influence between them in a simple choice task, especially if that influence would improve performance and this is explicitly explained to the participants. In contrast, here we show that human vision exhibits a specific kind of number blindness: performance on an area-choice task (which of these rectangles is larger?) is not improved by the addition of a perfectly correlated number signal (the larger one always has more dots on it) that creates equivalent density – even when explanations, reminders, and accurate feedback are given to the participants. This replicated across two experiments (N = 82, 122) with slightly different stimuli. Control analyses with brightness in Experiment 1 indicate that this is not a general resistance to the predicted cross-magnitude influence. This indicates that density, not number, is the primary independent perceptual dimension in human vision.

Given evolutionary pressures, human vision is not expected to redundantly estimate density, area, and number – within that trio, any two estimates can be used to derive the third estimate. Independent area estimation in the human visual system is well-documented (Yousif & Keil, 2019), but it remains unclear if density (Allïk & Tuulmets, 1991; Durgin, 1995, 2008; Morgan et al., 2014; Negen, 2023) or number (Anobile et al., 2014; Burr & Ross, 2008; Cicchini et al., 2016; DeSimone et al., 2020; Franconeri et al., 2009) is the other primary perceptual dimension. In other words, there is still controversy over whether human vision estimates number and area first, deriving density as number divided by area, versus estimating density and area first, deriving number as density times area.

This question is important because:

1. It impacts our basic view of how vision is organized and
2. Number perception is such a vital process.

First, a complete understanding of vision requires that we know what primary perceptual dimensions are available for visual judgements. Second, we should endeavour to fully understand how number perception functions because we know it is involved in many cognitive processes (Dehaene, 2011) and is related to high-priority outcomes in education, especially mathematics (Anobile et al., 2013; Halberda et al., 2008). Further discoveries about this topic could lead to important advances in education practice and related fields.

The present article aims to resolve this debate, to see if number or density is the primary independent perceptual dimension, by testing competing hypotheses from these accounts.

## Background

The study here is framed as helping to resolve between two modern accounts of number perception in human vision that have garnered recent support: number as primary or density as primary.

The number as primary account states that human vision somehow segments the objects in the attended visual field(s), estimates their number, and estimates the area in parallel. The estimation of number is described as direct, dedicated, rapid, and spontaneous (Cicchini et al., 2016). This does not mean that people can produce choices that totally disregard area (Leibovich & Henik, 2014), but it does theorize that the area calculations are independent until some point after the number estimate is found. Density can then only be later derived by division, often with great difficulty, high bias, and low precision. This view is supported in the literature, for example, by the claim that people spontaneously respond on the basis of number even when asked to attend to density alone (Cicchini et al., 2016).

The competing density as primary account states that human vision somehow segments the objects in the attended field(s), estimates their density, and estimates the area in parallel. This again does not mean that people can produce choices that totally disregard area (Leibovich & Henik, 2014), but it does theorize that the area calculations are independent until primary dimensions have been estimated. Number can then only be derived by multiplication with area, which can be very difficult for some observers (Halberda et al., 2008; Mazzocco et al., 2011) – though it is a highly practiced and salient skill in almost all extant cultures, so most tend be capable. This view is supported in the literature, for example, by the claim that the perception of density shows adaptation aftereffects (Durgin, 1995, 2008; Durgin & Huk, 1997); similar to the way that staring at something red and switching to something white makes it momentarily appear green, staring at a dense field of dots and switching to a reference field makes it momentarily appear less dense.

For our purpose here, we are disregarding a class of models that we might call degenerate, correlational, or confound-driven. This kind of model is essentially arguing that people (or some population) do not have any genuine perception of number. They might, for example, instead estimate the total area and make choices based on that in the hopes that area and number are correlated. This was a major debate regarding infancy (Clearfield & Mix, 1999) but not in adulthood. Please note that the density as primary account does not belong in this category because it is actually a valid algorithm for estimating number i.e. an accurate measurement of density times an accurate measurement of area will result in an accurate measurement of number; the density as primary account does not rely on any incidental correlations that could vary across stimulus sets. These degenerate / correlational / confound-driven models will not be discussed further here.

For our purpose here, we are also disregarding models that rely on unsegmented visual input. Imagine you have a field of dots. Imagine that you find pairs of nearby dots and draw a line connecting each pair, turning them into a shape more like a barbell. This procedure increases the number of unsegmented visual features (adding the lines) but it decreases the number of segmented visual objects (2 dots become 1 barbell). This procedure consistently decreases the perceived number of objects (Franconeri et al., 2009). We therefore focus on models (and corresponding stimuli) that rely on segmented visual features.

### Important clarifications

The ideas of area and density can have a surprising variety of meanings. In this text, the word area, unless otherwise specifically noted, refers to the area around an entire set of objects in a visual stimulus. The area of individual objects, or their summed area, is not something that will be particularly manipulated here. When experiments place objects into an unbounded void, it is not obvious exactly which ‘area’ will be attended in the task (e.g. convex hull, area of best fit circle, summed greatest distance along both cardinal axes, and so on). Here we manipulate the attended area by distributing objects randomly in a high contrast square of the intended area and making the area of that square relevant to performance on the task. Density is then defined here as the number of objects divided by the area. Note that density here is a feature of the entire set rather than something that varies continuously across the stimulus.

We should also be clear that this entire discussion is probably not relevant for very small sets i.e. those in the subitizing range. This range varies individually and developmentally but it is expected to be roughly 1–7 objects (Feigenson et al., 2004). In this lower range, accuracy is extremely high and Weber’s law ceases to apply (Revkin et al., 2008). This is thought to possibly be due to the ability to track each individual object in memory (Carey, 2009), a process that cannot apply to larger sets. The approach here will be to avoid stimuli with less than 9 objects. The results and discussion should therefore only be taken to apply to stimuli above the subitizing range.

However, the stimuli here are also designed to avoid a point of being so dense that they become hard to segment and thus necessarily processed as a texture. For example, one high-profile study found that their results were consistent with the density as primary account when there were up to 160 low-contrast dots in a small display area (Cicchini et al., 2016), despite providing several arguments that number must be primary in less dense displays. The method here will re-use 12 dots as the standard to avoid any similar issues.

### Logic of the present study

The central question here, whether density or number is primary, has remained unresolved for so long because the logical interlocking of density, area, and number precludes the effective application of many basic research designs. If you have two displays of dots that differ in number, they must necessarily also differ in density, area, or both. If you have two displays of dots that differ in density, they must necessarily also differ in number, area, or both. There has been extensive controversy about the best ways to work around this (DeWind et al., 2015; Leibovich & Henik, 2014; Park, 2022; Piantadosi, 2016; Szűcs et al., 2013; Whalen et al., 1999). Despite all of this work, research has never found any way to obtain participant responses that are purely a function of only one magnitude (Leibovich et al., 2017). When we ask participants to judge any given magnitude, we always expect some kind of influence from at least one other magnitude.

**Fig. 1:**
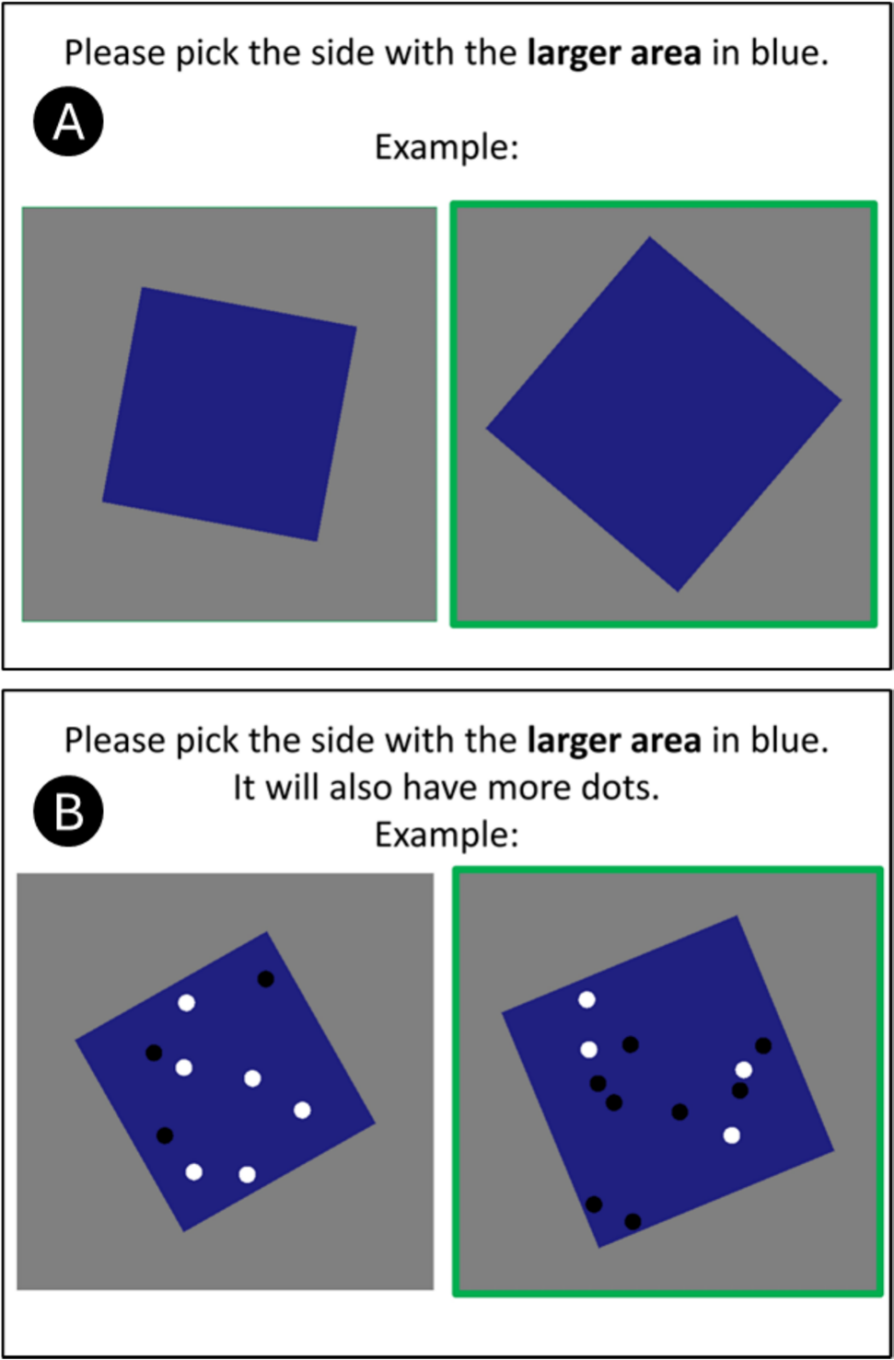
Key methods. (**A**) Instructions and example stimuli for the area-only trials. (**B**) Instructions and example stimuli for the area & number trials. The ratio of the number of dots (here 9:12) was equal to the ratio of the areas, holding the density equivalent

In contrast to previous research, which treats cross-magnitude influence as a nuisance to be reduced and corrected, the present study instead levers cross-magnitude influence as a test for perceptual primacy. The baseline task here is a simple choice task where participants are asked to say which of two stimuli is greater in area (area-only trials; Fig. 1A). The key manipulation is that another kind of trial also provides a congruent numeric difference (area & number trials; Fig. 1B) with an exact match between the area ratio and the numeric ratio. The two accounts then make different predictions. If number is primary, adding this numeric difference should aid performance. If density is primary, it should not aid performance.

To understand the logic of this, it will help to draw an analogy to simple survey research. Suppose you are interested in the approval rate for a local politician. Suppose you already have one group of survey responses. If you get a second independent group of participants, you can improve your estimate by averaging the results from the two groups. In contrast, imagine you instead just made a photocopy of all the responses from the first group – except some responses were lost or flipped due to machine errors. Averaging together the results from the first group and the photocopies is not going to lead to a better estimate. It might actually make things worse. This is analogous to what we should expect when comparing the area-only trials here against the area & number trials: adding the numeric difference is like a new group if number is primary but it is like a photocopy if density is primary.

This should become clearer when we trace the process of perception under each account. If number is a primary perceptual dimension, then the numeric difference estimate and the area difference estimate are independent in the area & number trials. Errors will tend to average out much like averaging two independent participant groups. If density is primary, then each numeric estimate is derived by multiplying a density estimate and an area estimate. This means that our numeric difference estimate is just a copy of our area difference estimate – except it is also adjusted by the density estimates, which should be equivalent on average (since density is actually equivalent in the present design) but will add some noise as well. This creates a numeric difference estimate that cannot help for the same reason that photocopying survey results does not help. We need independence in the signals for averaging to reduce noise, which is provided in the area & number trials if number perception is primary but not if density is primary.

The following two experiments test these competing hypotheses. The first follows recent research (Cicchini et al., 2016) using a random mix of black and white dots. The second uses white dots that are surrounded by black rings to make sure that the results replicate when the effect of brightness is attenuated.

## Experiment 1

The hypothesis for this experiment was that performance will be better in the area & number trials compared to the area-only trials. If true, this points towards number as a primary perceptual dimension. If not, this points towards density as a primary perceptual dimension. The following text also reports a post-hoc test that uses brightness in place of number, presented as a check on the validity of the main method and analysis.

### Method

**Fig. 2:**
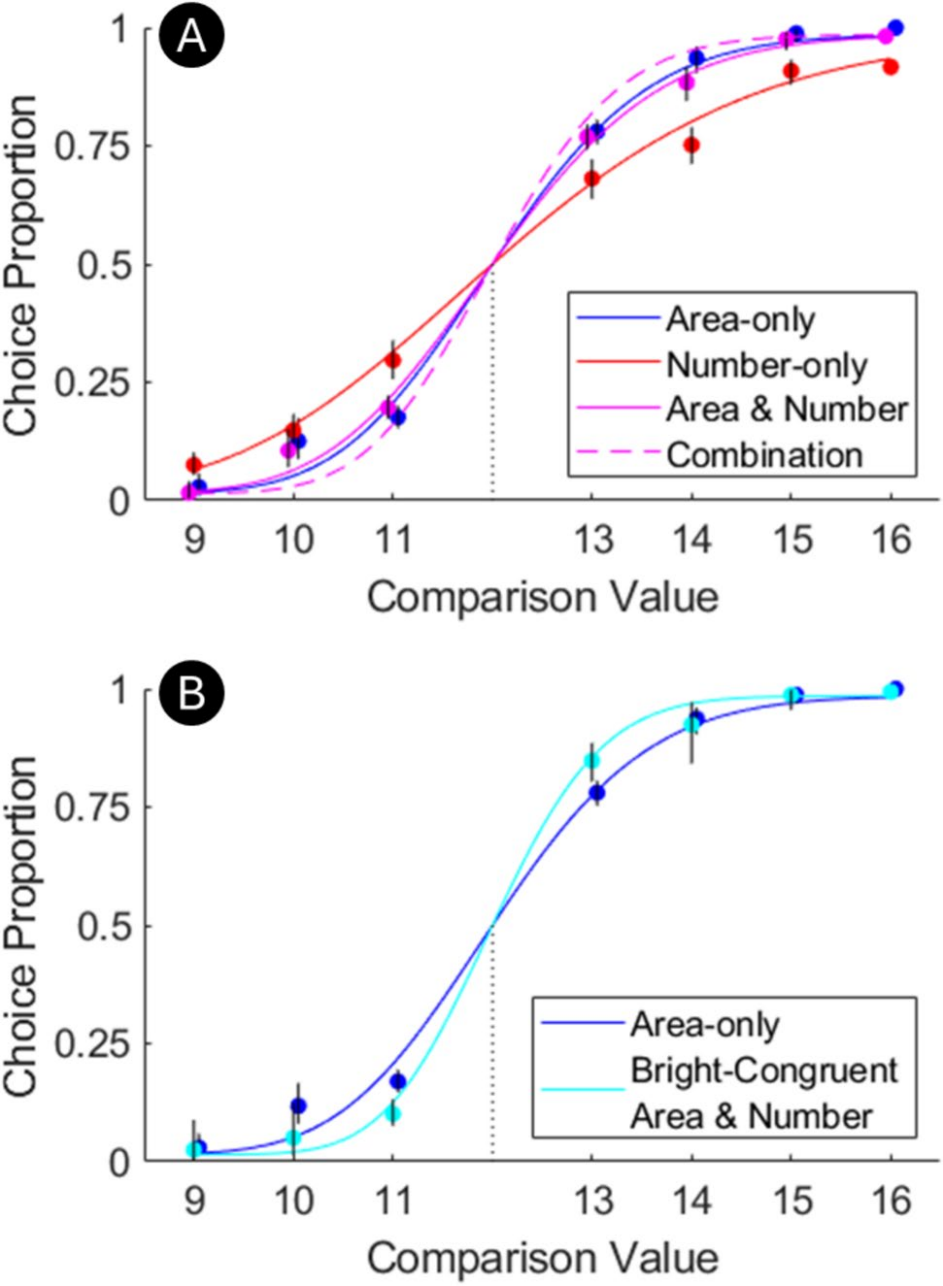
Key results. (**A**) Performance was not better in the area & number trials than the area-only trials. It also fell short of what we would predict from their combination, shown as a dashed line. Lines are median fit and dots are aggregate performance, marked with 95% confidence intervals. The standard was always 12. (**B**) Bright-congruent area & number trials are the selected portion of area & number trials where the side with larger area had higher average brightness due to randomly having a greater percentage of white dots. Performance indicates that cross-magnitude influence is present in this experiment

A pre-registration containing all stimuli, the code for running the experiment, and the code for the main analysis (Fig. 2A) including the exclusion rules is at 10.17605/OSF.IO/E42QN. The related OSF project also has all of the data and code, including the post-hoc analysis.

#### Participants

82 Participants were ultimately included (reported age mean = 30.12 years, SD = 9.46, with 6 preferring not to say; 46 male, 30 female, 1 non-binary, 5 prefer not to say). They were recruited on Prolific and screened for normal or corrected-to-normal vision and fluency in English. Six more were lost to technical errors and two were excluded because they did not perform significantly above chance. Observations were excluded if they were more than three times larger than the median, as described in the pre-registration, which happened to be necessary for the post-hoc analysis in Fig. 2B (resulting in N = 79) but not the pre-registered analysis in Fig. 2A. Participants were paid £1.35 for participation (approx. £10/hour). Ethics approval was given by Liverpool John Moores University Research Ethics Committee (Ref: 22/PSY/027). Informed consent was obtained in written form from all participants.

Sampling continued until a single obligatory stopping point was reached in a Bayesian framework. Note that this scheme shares some surface features with questionable optional stopping practices, but it is distinct and it does not lead to the same issues. The key comparison was the weber fractions for area versus area & number trials, instantiated as a one-sided Bayesian t-test with area & number being better. The stopping rule was that data collection stops when we either have strong evidence that there is an effect (BF_10_ > 20) or strong evidence that there is not an effect (BF_01_ > 20), but continues otherwise. The sample began with 30 participants, then increased in blocks of 5 until the rule was triggered, also in accordance with the pre-registration.

#### Design

This was a two-way within-subject experimental design. The first factor was trial type: area-only, number-only, or area & number. The second was the ratio of the comparison to the standard: 9/12, 10/12, 11/12, 13/12, 14/12, 15/12, or 16/12.

#### Procedure

There were three blocks of trials in random order. Each began with an instruction slide (Fig. 1). Area-only and area & number trials asked the participant to choose the side with larger area. Number trials asked the participant to choose the side with more dots. Each block began with 7 training trials (not analyzed) and followed with 42 testing trials.

On each trial, a fixation cross was shown for 1s. The standard and comparison were presented side-by-side for 500ms. The participant then clicked where one of the two stimuli was shown (no time limit). The correct one was surrounded by a green border. If the participant chose correctly, this lasted for 0.25 seconds. If not, it lasted for 2s. This creates a small but salient economic incentive as participants on this platform are paid for completion of each study, which means they make the best hourly wage by avoiding time penalties. This should give participants a motivation to perform better on the area & number trials if they can.

For the number-only trials, the ratio of the comparison to the standard was evenly distributed with 6 testing trials per possible ratio. For the other two, the lowest two ratios had 3 testing trials, the 11/12 and 13/12 ratios had 12 testing trials, and the highest three ratios had 4 testing trials. For all trial types, the training trials were evenly distributed across ratios.

#### Stimuli

For area-only trials, a mid blue square (h = 2/3, s = 1/2, v = 1/2) was placed at a random rotation in the center of a mid grey void. The standard was 12 units where 32 units is the area of the void. A reminder text read "larger area".

For number-only trials, a set of dots were placed in random non-overlapping positions inside a mid grey void. The standard was 12 dots. These dots were also constrained by a square at a random rotation but that square was not drawn explicitly. For the standards, that undrawn square was also 12 units of area. For half of trials, the comparison/standard ratio was the same for both area and number. For the other half, the area ratio was the inverse of the number ratio. Please note that this makes for a kind of lower bound on the precision of number perception as their will be some interference from area. Each dot was either white or black. The percentage of each color was chosen randomly between 20% and 80%. A reminder text read "more dots".

For area & number trials, every trial re-used a blue square from an area-only trial. Dots were drawn onto it with the same constraints on colors as above. The standard had 12 dots and 12 units of area. The comparisons had the same ratio to the standard in both area and number. This means that density was constant, which is necessary for the two competing accounts here to make different predictions. A reminder text read "larger area (will also have more dots)".

#### Pre-registered analysis

Weber fractions were fitted with a typical signal detection model:

$$\text{P}\left(c\right)=\Phi \left(\mu =c-s,\sigma =\sqrt{{\left(ws\right)}^{2}+{\left(wc\right)}^{2}}\right)\times .97+.015$$

where P(c) is the probability of choosing the comparison stimulus, Φ is the normal cumulative distribution function, *s* is the magnitude of the standard, *c* is the magnitude of the comparison, and *w* is the fitted weber fraction. To avoid degenerate w = 0 fits, a half-trial was added to every participant where the comparison/standard ratio was 11/12 and the comparison stimulus was chosen. The combination prediction was ${\left({w}_{a}^{-2}+{w}_{n}^{-2}\right)}^{-1/2}$ where ${w}_{a}$ is the weber fraction for area-only and ${w}_{n}$ is the weber fraction for number-only (Rohde et al., 2016). One-sided t-tests were used for all analyses (area worse than area & number; area & number worse than combination prediction). The Bayesian t-tests used a Cauchy prior with a scale of 0.707.

#### Post-hoc analysis

The post-hoc analysis in Figure 2B uses a selected portion of the area & number trials. It looks specifically at trials where the side with greater area happened to be brighter due to randomly being drawn with a greater percentage of white dots. (Note that this is not every trial with more white dots on the side with greater area.) These trials are referred to below as *bright-congruent area & number trials*. They were fit with the same parameter estimation described above and were subject to the same exclusion rules. The analysis compares them to the area-only trials to check that we can observe normal cross-magnitude influences in this experiment.

## Results

Descriptive statistics are given in Table 1. Some skew is present (mean > median) but it is small enough to not be an issue for a t-test. In contrast to the hypothesis, performance was no better in the area & number trials than the area-only trials (Fig. 2A; t(81) = −2.00, p = .976, d = -.22, 95% CI: -.001 to -.439, BF_01_ = 23.6). It was also significantly worse than we would expect by combining their performance on the area-only and number-only trials under the assumption of independent perception (t(81) = 5.54, p < .001, d = .61, 95% CI: .375 to .847, BF_10_ = 7.7x10^4^). By the pre-registered logic, this points towards density as the primary perceptual dimension.

**Table 1:** Descriptive Statistics for all analysed Weber fractions in Experiment 1

Trial Type	Median	Mean	Standard Dev.
Area-only	.0723	.0743	.0288
Number-only	.123	.132	.0457
Area & Number	.0782	.0830	.0365
*Combination*	.0595	.0612	.0209
*Bright-Congruent Area & Number*	.0509	.0603	.0317

Note. Rows in italics are not independent trial types. Combination is derived from area-only and number-only trials (see *Pre-registered Analysis* above). Bright-congruent area & number trials are a selected portion of area & number trials (see *Post-hoc Analysis* above)

Post-hoc analysis was run to be as sure as possible that the area cue in this experiment was not somehow resistant to cross-magnitude influence. Performance was better for bright-congruent area & number trials than area-only trials (Fig. 2B; t(78) = 2.86, p = .005, d = .322, 95% CI: .095 to .547, BF_10_ = 10.6). In other words, brightness did show the predicted cross-magnitude influence onto area.

### Discussion

These results point strongly towards the density as primary account. This account correctly predicted that the area & number performance would not be better than the area-only performance. This is confirmed with a positive Bayesian finding (BF_01_ = 23.6) and a non-significant p-value in a large sample (p = .976, N = 82). It also correctly predicted that area & number performance would be worse than their combination. Post-hoc analyses further suggest that there was not some novel resistance to cross-magnitude influence onto the area cue. This is shown by the influence of brightness in the bright-congruent area & number trials. This is despite the fact that number was a valid cue, which was highlighted to participants, while brightness was not a valid cue and was not highlighted to participants.

With all of that said, once these results are known it becomes possible to see a variation on the experiment that could be an even stronger test of the hypothesis. The stimuli here were based on previous experiments that favoured number as primary, especially a recent one in a high profile journal (Cicchini et al., 2016). This meant using a random percentage of black and white dots, which creates the incidental brightness cue. Now that we know that this does not show an effect of the added number signal, but that the varying brightness does influence performance, we can adjust the experiment to attenuate brightness differences and see if a different pattern emerges. Experiment 2 fills this role.

## Experiment 2

**Fig. 3:**
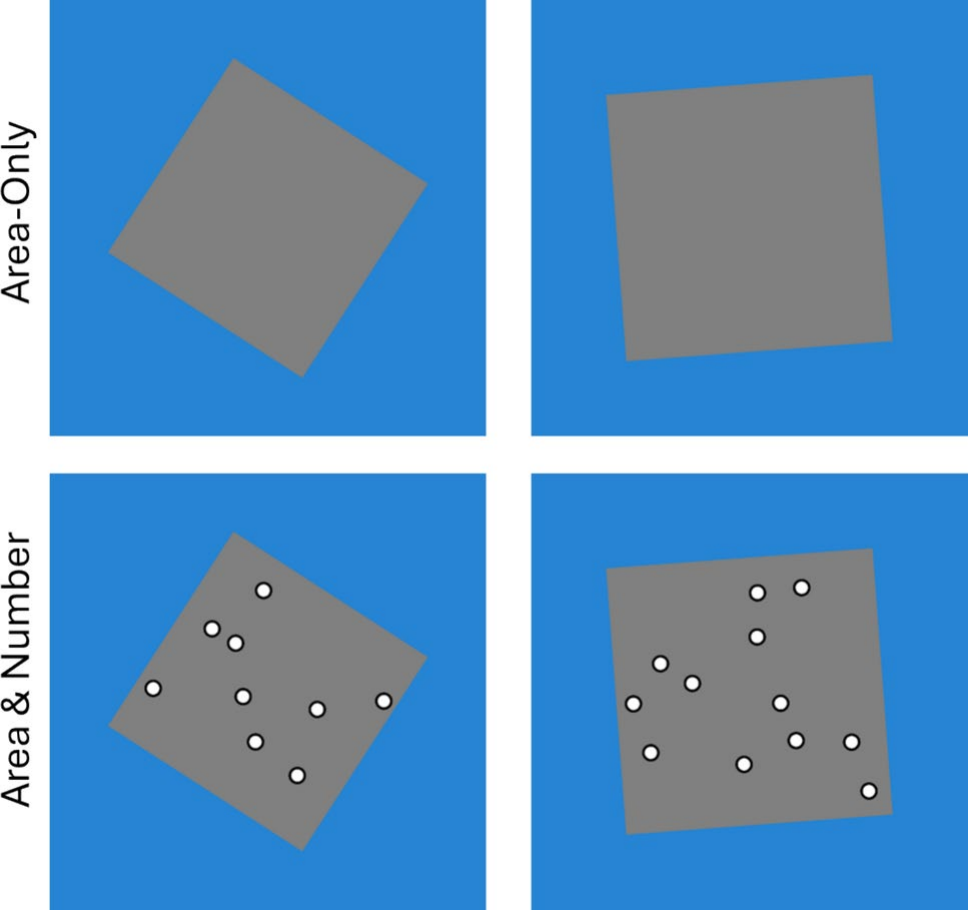
Revised Stimuli for Experiment 2. Note that all stimuli now have similar overall brightness because the blue and grey are both near the middle of the scale and the dots have equal area for their black and white parts

The point of Experiment 2 was to see if the pattern of results from Experiment 1 holds when the variation in brightness is attenuated. The most important goal here is to minimize the impact on the brightness of the squares when the dots are added, thus minimizing any effect on the crucial area-only versus area & number comparison. This was done by changing the squares to a mid grey (R = .5, G = .5, B = .5) and changing the dots into white circles surrounded by black rings such that each has equal area (Fig. 3). This means that every square should have very similar brightness, both with and without the dots. This has the favourable side effect of also making the dots maximally visible.

The second most important goal is to generally reduce the variance of the brightness in the whole stimulus set. To do this, the number-only trials were given the same mid grey as their whole background. The area-only and area & number trials were given a new blue-green background color (R = 38/255, G = 132/255, B = 212/255) that is as close as possible in terms of average RGB and the lightness dimension in CIELAB space (about 53% for both). This means that all stimuli should have very similar total brightness.

### Method

A pre-registration containing all stimuli, the code for running the experiment, and the code for the analysis including the exclusion rules is at https://osf.io/u5276. The related OSF project also has all of the data. The design, procedure, and pre-registered analysis are all the same as Experiment 1.

#### Participants

122 Participants were ultimately included (reported age mean = 36.79 years, SD = 13.40, with 25 preferring not to say; 61 male, 36 female, 25 prefer not to say). They were recruited on Prolific and screened for normal or corrected-to-normal vision and fluency in English. Four more were lost to technical errors, thirteen more were excluded because they did not perform significantly above chance, and one more was excluded because a fitted Weber fraction was more than three times larger than the median. Participants were paid £1.35 for participation (approx. £10/hour). Ethics approval was given by Liverpool John Moores University Research Ethics Committee (Ref: 22/PSY/027). Informed consent was obtained in written form from all participants. Sampling continued with the same rule as Experiment 1 (until BF_01_ > 20 or BF_10_ > 20 for the t-test on fitted Weber fractions, comparing area-only trials versus the area & number trials).

#### Stimuli

Stimuli were the same as Experiment 1 except for the changes in colors and the changes in the dots. The squares for area-only and area & number trials were changed to a mid grey (R = .5, B = .5, G = .5). Their backgrounds were changed to a lightness-matched blue-green (R = 38/255, G = 132/255, B = 212/255). The number-only just used a solid mid grey background. The dots themselves were changed to white dots surrounded by black rings of equal area.

### Results

**Fig. 4:**
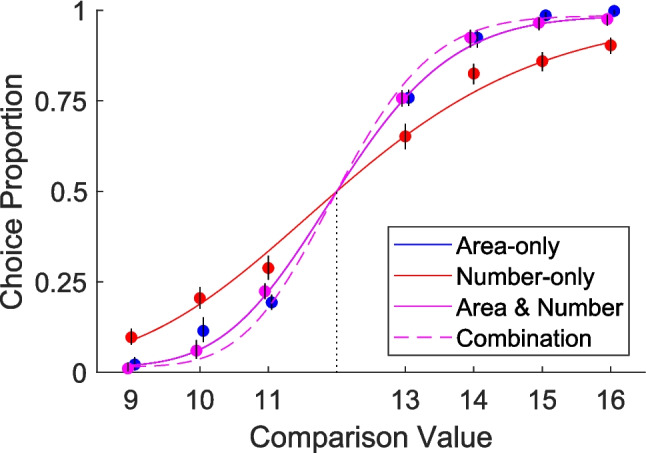
Key results. Results replicated from Experiment 1. Performance was not better in the area & number trials than the area-only trials. It also fell short of what we would predict from their combination, shown as a dashed line. Lines are median fit and dots are aggregate performance, marked with 95% confidence intervals. The standard was always 12

Results replicated Experiment 1. Descriptive statistics are given in Table 2. Performance was no better in the area & number trials than the area-only trials (Fig. 4; t(121) = −1.17, p = .877, d = -.106, 95% CI: -.283 to .0725, BF_01_ = 20.4). It was also significantly worse than we would expect by combining their performance on the area-only and number-only trials under the assumption of independent perception (t(121) = 5.33, *p* < .001, d = .483, 95% CI: .294 to .6694, BF_10_ = 5.4x10^4^). By the pre-registered logic, this again points towards density as the primary perceptual dimension.

**Table 2:** Descriptive Statistics for all analysed Weber fractions in Experiment 2

Trial Type	Median	Mean	Standard Deviation
Area-only	.0777	.0785	.0287
Number-only	.139	.146	.0570
Area & Number	.0778	.0828	.0328
*Combination*	.0642	.0651	.0203

Note. Final row in italics is not an independent trial type. Combination is derived from area-only and number-only trials (see *Pre-registered Analysis* above)

### Discussion

Experiment 2 replicated Experiment 1 even after brightness differences were attenuated. This suggests that the results from Experiment 1 were not due to issues with interfering brightness or dot visibility. This in turn reinforces the conclusion that number does not create cross-magnitude influence with area when density is constant, confirming a prediction from the account where density perception is primary.

## General Discussion

In these specific circumstances, participants were not able to improve performance by using the differences in the number of dots. If area and number are both primary independent dimensions of perception, then this task should have created the usual cross-magnitude influence. This influence would help because the area and number were always congruent. No such improvement was observed. This is despite samples of 82 and 122 participants. This is also despite explicit instructions and feedback. It is also confirmed by positive Bayesian findings (BF_01_ = 23.6, 20.4) and significant differences between the combination predictions versus the area & number performance observations. Post-hoc analyses in Experiment 1 also showed that brightness improved performance when it was congruent, despite the fact that brightness was not part of the instructions and was not even a valid cue. This suggests that the experiment here is not an unreasonable test for a primary perceptual dimension to pass. In contrast, the results here are exactly as predicted under the account that density perception is primary. As described in detail in the introduction and pre-registration, we do not expect the numeric difference to help if perception is deriving number estimates from area estimates since this means they are not independent. All of this points towards density perception as primary, alongside area, with our number estimates derived as density times area.

The key innovation here is that the basic method tries to work with cross-magnitude influence rather than against it, which may explain some contrasting results in the literature. The logic here never requires any specific correction for cross-magnitude influence. In the area-only trials, there is no difference in either density or number to correct. In the area & number trials, we are interested in the influence. Even in the number-only trials, we can be satisfied with an uncorrected lower bound on precision as they are only used for the combination prediction, which already returned a difference from the observed combination, so any correction would just increase the reported effects. In the available literature, the method for creating cross-magnitude corrections varies from study to study (Leibovich et al., 2017) and it is not obvious how much each result depends on the exact choice of correction. These and many other technical issues (e.g. the exact instructions, the role of out-of-lab perceptual learning, the use/avoidance of feedback) are all avoided by using cross-magnitude influence to our advantage.

Readers who are very familiar with this research area will also recognize that the standards were chosen here to avoid the potential dissociation in mechanisms at different densities (Anobile et al., 2014; Cicchini et al., 2016; Zimmermann & Fink, 2016). These theories propose that number perception does (or at least could) depend on density perception at higher densities, but not in sparser displays. The highest density used here put just 16 dots on one half of the screen, which should make it clear that sparse densities are being addressed directly. Future research could of course repeat the method here with higher densities if that became theoretically useful.

This finding also reinforces the broader view that human perception does deal with number but not with the specific algorithm of serial counting (Carey, 2009; Feigenson et al., 2004). For example, if we count out loud one by one, the errors follow a law of $\sigma =c\sqrt{n}$ (standard deviation, constant, number of items to be counted), whereas estimates follow a $\sigma =cn$ law when verbal counting is blocked (Cordes et al., 2001), suggesting a dissociation. In other words, evolved number estimation algorithms and taught counting procedures are fundamentally different approaches. The results here add detail to this conclusion, suggesting that human vision has evolved a kind of density-area mechanism for estimating number instead of a serial counting process.

To conclude, the existence of the specific number blindness described here indicates that human vision estimates density and area first, then later estimates number via multiplication. This still means that number is something that human vision does estimate, but it is not a primary dimension for vision, the estimate is not a direct estimate, and the way the estimate is found bears virtually no resemblance to the way we teach young children to determine number via counting. Human vision has instead evolved an indirect system to estimate number, first estimating density and later deriving number.

## Data Availability

All data and materials are available at https://osf.io/rtgk4/

## Code availability

All code is available at https://osf.io/rtgk4/
